# Anemia in children aged 6 to 59 months attending a quaternary health facility in Maputo, Mozambique

**DOI:** 10.1093/eurpub/ckac129.697

**Published:** 2022-10-25

**Authors:** R Maulide Cane, Y Keita, L Varandas, I Craveiro

**Affiliations:** 1 Instituto Nacional de Saude, National Institute of Health, MoH, Maputo, Mozambique; 2 Global Health and Tropical Medicine, Instituto de Higiene e Medicina Tropical, UNL, Lisbon, Portugal; 3 GIZ/C4N-NIPN, Clinton Health Access Initiative, Bamako, Mali; 4 Hospital CUF, Descobertas, Lisbon, Portugal; 5 Faculdade de Ciências Medicas, NOVA Medical School, UNL, Lisbon, Portugal

## Abstract

**Background:**

Globally, anemia prevails as a public health issue, being also a concern in Mozambique, where about two-thirds of children 6-59 months of age are affected by anemia. We carried out this study to estimate anemia prevalence and evaluate structural determinants and hematological parameters association among children aged 6 to 59 months attending pediatric inpatient and outpatient services in a Quaternary Health Facility in Maputo City Province, Mozambique.

**Methods:**

From August 2021 to January 2022, we conducted a cross-sectional study at the ‘Maputo Central Hospital’ where we collected data from 397 inpatients or outpatients who attended pediatric consultations. The cut-off values for anemia were: mild (10g/dL≤Hb≤10.9g/dL), moderate (7g/dL≤Hb≤9.9g/dL), severe (Hb < 7.0g/dL). We used SPSS 28.0 software to perform descriptive analyses and Chi-Square tests.

**Results:**

Our preliminary findings show that the total rate of positive cases was 30.0% moderate anemia (119/397), 23.9% mild anemia (95/397), and 7.3% severe anemia (29/397). Anemia frequencies were higher in male patients unregarding the type (54.2% moderate, 62.1% mild, 67.9% severe). Anemia prevalence was higher among children aged 24-59 months (41.2% moderate, 47.4% mild, 51.7% severe; p < 0.05). The rate of all anemia types was higher in children from rural areas and Maputo City province relative to those from urban areas and other country provinces. The level of education of the child's companion to the consultations was associated with anemia (p ≤ 0,05), with higher rates observed in secondary level education. We observed no association between iron or serum ferritin values to anemia.

**Key messages:**

• Children aged 24-59 months, children from rural areas, and who are male are more vulnerable to suffering anemia than their peers, thus needing more monitoring during their growth.

• Nutritional-anemia-specific interventions targeting the first 1000 days of life may be helpful to its reduction in children.

